# Layered HARQ Design for LDPC-Based Multi-Level Coded Modulation

**DOI:** 10.3390/e27060629

**Published:** 2025-06-13

**Authors:** Yuejun Wei, Yue Chen, Chunqi Chen, Bin Xia, Liandong Wang

**Affiliations:** 1 School of Computer and Information Engineering, Shanghai Polytechnic University, Shanghai 201209, China; 20231513141@sspu.edu.cn (Y.C.); ldwang@sspu.edu.cn (L.W.); 2 Department of Electronic Engineering, Shanghai Jiao Tong University, Shanghai 200240, China; ccq123@sjtu.edu.cn (C.C.); bxia@sjtu.edu.cn (B.X.)

**Keywords:** low-density parity check, multi-level coded modulation, hybrid automatic repeat request, chase combing, incremental redundancy, bit rearrangement

## Abstract

Multi-level coded modulation (MLCM) enhances data transmission by allocating error correction more effectively to bits with higher error probabilities, thus optimizing redundancy and improving performance. Despite MLCM’s advantages over traditional bit-interleaved coded modulation (BICM) systems in certain scenarios, its integration with hybrid automatic repeat request (HARQ) systems remains underexplored. HARQ, which combines the benefits of forward error correction (FEC) and automatic repeat request (ARQ), significantly increases resilience to interference and fading, enhancing overall system reliability. This paper bridges the gap by integrating HARQ techniques into the MLCM framework, which was specifically adapted to the layered nature of MLCM. We present tailored hybrid retransmission strategies for each layer of MLCM, demonstrating substantial gains in retransmission efficiency and overall transmission performance.

## 1. Introduction

The bit-interleaved coded modulation (BICM) scheme, which is designed to enhance the transmission performance of data under fading channels by introducing a bit interleaver [1], was proposed in 1992. It is closer to the optimal coded modulation scheme in terms of performance under fading channels, making it more suitable for practical applications in fading channels [2]. However, the shortcoming of BICM is its high computational complexity, especially at high code rates or high modulation orders, where the decoding delay and hardware implementation complexity increase significantly. Furthermore, signal superposition in bidirectional relay scenarios can disrupt Gray mapping and increase the bit error rate, while layered constellation design can improve performance by increasing the Euclidean distance of key bits [3]. In the existing layered scheme, multi-level coded modulation (MLCM) decoding complexity is lower, and it could be more flexible in combining different modulation methods and coding schemes to achieve better performance, which is easy to implement in hardware and more suitable for future complex application scenarios, such as future unmanned aerial vehicle, Industry 5.0, and space and deep-sea tourism.

MLCM is a communication technique that combines multi-level coding and modulation. The concept of joint coding and modulation design first appeared in 1974 [4], and it also laid the foundation for the subsequent MLC scheme [5]. It optimizes the overall performance using component codes with different rates to protect the information bits in layers. This scheme of layering each data point separately for coding and modulation can reduce the complexity of coding and decoding under higher-order modulation, and it has attracted the attention of a wide range of scholars in recent years [6,7,8].

It divides the data into bit streams with different signal-to-noise ratios (SNRs). Each bit stream is assigned a different error correction code. The essence is that, when the bit sequence is mapped to a Quadrature Amplitude Modulation (QAM) constellation point, the bit data with different SNRs will not be protected to the same extent. Since its development, MLCM has become one of the key technologies in the field of communication. A multi-level coding technique based on spatially coupled coding has been proposed that proves that MLCM is more efficient than the traditional FEC configuration of BICM, and its efficiency tends to increase with an increase in the modulation order [9]. The rate diversity technique proposed by Chen et al. can significantly improve performance and adapt to channel differences by allocating different bit rates under different channel conditions [10], thus providing an innovative direction for the research in this paper.

Due to channel noise and other interferences, it is also inevitable that MLCM will suffer from miscommunication in the process of transmitting data, resulting in packet loss.

HARQ is one of the main means of improving the reliability of communication systems today. Many scholars have analyzed the performance of the HARQ protocol [11,12] from the perspective of information theory; in order to improve the system throughput, some optimization methods with applicability [13,14] and various adaptive incremental redundancy schemes have been proposed [15,16,17], and different algorithms have also been used to solve the problem of inflexibility in retransmission [18], which can be seen as there being a wide range to HARQ’s technical applications and to the maturity of the technological development.

The HARQ approach effectively combines the benefits of FEC and ARQ while mitigating their respective drawbacks. It can increase link throughput, better overcome the time-varying and error-prone wireless channel conditions, bolster anti-jamming and anti-fading capabilities, and improve overall data transmission system effectiveness and reliability, rendering it a more viable solution. There also exists existing technical results that demonstrate the incorporation of HARQ technology in MLCM to enhance the performance of MLCM systems [19].

However, for the whole research field, there are few schemes combining HARQ and MLCM in existing technology, and the integration of the two technologies is not mature. The application of HARQ technology in MLCM systems is often neglected, mainly because of the different coding methods used in each transmission layer and the conditions of use of HARQ, which cannot be generalized to each layer directly.

In this study, a flexible HARQ and MLCM co-design framework is proposed to integrate HARQ technology into MLCM systems. Considering the inherent structural characteristics of hierarchical coding and modulation architectures, the transmission layer of MLCM adopts different coding methods depending on the bit streams. In addition, a bit retransmission mechanism is added to the transport layer using the CC method to further enhance the transmission gain. The MLCM-HARQ scheme is simulated under the MLCM hierarchical structure with different code rates, and the gain can be improved by about 3–5 dB at different code rates.

## 2. Related Work

Existing solutions to improve reliability in the field of communication mainly involve the introduction of error control techniques, which mainly include ARQ, FEC, and HARQ [20]. Error control technology is that the sender side will resend data when the system transmits data in error. ARQ technology is simply retransmitting data. However, the system will not allow unlimited transmissions and will set the maximum number of retransmissions in advance. It is also possible for the system to compare the content of several retransmissions and check the content after they have been combined in order to find the correct ones as soon as possible.

While the ARQ error control mechanism provides high transmission reliability and is relatively simple, the throughput of the ARQ scheme decreases rapidly and the latency may rise too high as the channel error rate increases. This prevents it from being used in some highly sensitive systems. On the other hand, the system using FEC has constant throughput and is not affected by the channel error rate though. However, it is not highly reliable and has requirements for error correction code performance. And when used in special scenarios, FEC codes must minimize the use of redundant bits while ensuring error correction performance [21]. In order to overcome their respective drawbacks, it is necessary to combine these two error control schemes appropriately.

HARQ is a combination of FEC and ARQ techniques to improve transmission reliability through retransmission and merging mechanisms. This approach corrects for frequent errors and thus reduces the average number of retransmissions. When the frequency of errors is low, each retransmission carries some redundant information to aid in packet detection. FEC is first used to correct some of the errors at the receiver, and it relies on error detection to detect the remaining errors. If the remaining errors cannot be corrected, the packets that cannot be corrected are discarded and a request is made to the transmitter to retransmit the packet. The HARQ technique with soft merging is often classified into two types depending on whether the retransmitted bits are the same as the initial ones: the CC method and the IR method [22,23].

In modern communication systems, such as 4G LTE and 5G NR, the CC method is widely used due to its simple implementation and stable performance. The initial transmission and each retransmission of CC are exactly the same, and the protocol complexity and receiver-side storage requirements are greatly reduced. Its core idea is to improve the SNR of the received signal by combining the same packets transmitted multiple times, thus improving the decoding success rate [24].

The CC method sends the same data at the initial and retransmission times, thus increasing the probability of correct decoding, while the IR method sends different redundant versions step by step, which reduces the channel coding rate and improves the coding gain. The spectral efficiency of the CC method is lower than the IR method because the IR method is able to introduce new redundant information and has a higher decoding success rate for the same number of retransmissions. However, the CC method has lower complexity and storage requirements so it is more advantageous in low-latency and high-reliability scenarios. The IR method not only obtains the energy gain and diversity gain, but it also obtains the additional net coding gain due to the code rate reduction, which has good application performance in different scenarios [25,26]. However, the code rate compatibility characteristics of channel coding must be considered.

It can be seen that these methods have their own suitable areas, and this characteristic matches the flexible layered structure of MLCM. In this paper, according to the characteristics of different coding layers, we chose the respective suitable CC or IR methods, which made the integration of MLCM and HARQ technology more complete and universal.

## 3. The MLCM-HARQ Schemes

### 3.1. MLCM Layering Scheme

The choice of forward error correction code type is crucial for MLCM. In the mid-1990s, MacKay et al. discovered that Low-Density Parity-Check (LDPC) codes approximate the channel capacity under iterative decoding algorithms with a low complexity to the compiled code, which triggered a research boom [27]. LDPC codes have become one of the main coding schemes in 5G communication systems. At long code lengths, LDPC codes are especially close to the Shannon limit of error correction performance. LDPC coding can dynamically adjust the code rate by changing the density of the checksum matrix, and it can be perfectly adapted to the differentiated protection requirements of each coding layer in MLCM. The LDPC coding hardware has been highly optimized, and it can be directly integrated into the processing link of MLCM, which reduces the development cost of the system.

Bose–Chaudhuri–Hocquenghem (BCH) codes, although less frequently used in MLCM systems than LDPC codes, can still be useful in specific scenarios. The BCH algorithm can be quickly implemented in hardware by the look-up table method. Compared with the iterative decoding of LDPC, BCH codes consume less energy for short code lengths (n<1000). Its error correction efficiency is close to the theoretical optimum, which complements the excellent performance of LDPC codes with long code lengths. BCH codes avoid the redundancy overhead of LDPC long codes if the data block of a coding layer is small in MLCM. Moreover, compared with LDPC decoding, BCH codes have negligible decoding complexity in some specific code length and code rate cases [28,29].

As mentioned above, considering the error correction capability, decoding complexity, and matching with channel characteristics, this scheme was illustrated using a three-layer structured MLCM system as an example. The specific coding layer was divided by the bit SNR. Bit streams with a low SNR were encoded using error correction codes with high complexity but high error correction capability. Bit streams with a medium SNR were encoded with error correction codes of low complexity but with some error correction capability. Bit streams with the highest SNR may not even be encoded. In this scheme, the lowest SNR was coded by LDPC coding. The bits with medium SNRs were coded using BCH coding with lower complexity. And the bits with the highest SNRs were categorized as no coding (NC) to reduce the overall complexity of the system without affecting the reliability.

**Figure 1:**
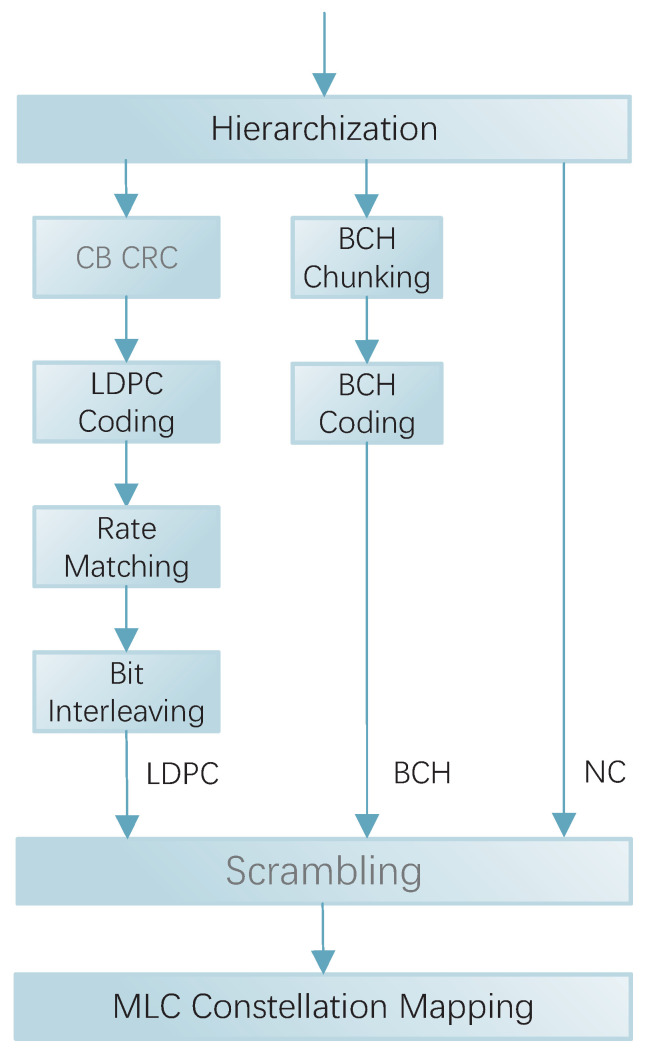
MCLM transmitter schematic.

The LDPC layer acts as the core layer and contributes most of the gain to the overall MLCM, as shown in Figure 1. The bit stream at LDPC layer goes through Code Block Cyclic Redundancy Check (CB CRC), LDPC coding, Rate Matching and Bit Interleaving [30]. Firstly, to ensure that transmission errors can be detected at the receiver, CRC check bits are added to the input Transport Block (TB). A 24-bit CRC is used in 5G NR, which has a generating polynomial as follows:

(1) $$\begin{array}{cc} g_{\mathrm{CRC}24A}\left(D\right)= & \;D^{24}+D^{23}+D^{18}+D^{17}+D^{14}+D^{11}+D^{10}\\ \; & \;+D^{7}+D^{6}+D^{5}+D^{4}+D^{3}+D+1. \end{array}$$

If the length of the TB exceeds the maximum input limit of the LDPC encoder, it needs to be partitioned into multiple CBs, and CRCs are appended to each CB. Let the TB length be *B* and the maximum code block input length be $K_{\max}$. Then, the number of code blocks after splitting is as follows:

(2) $$C=\left\lceil \frac{B+L_{\mathrm{CRC}}}{K_{\max}-L_{\mathrm{CRC}}}\right\rceil ,L_{\mathrm{CRC}}=24.$$

The effective message length of each code block after segmentation is

(3) $$K=\frac{B+C\cdot L_{\mathrm{CRC}}}{C}.$$

**Figure 2:**
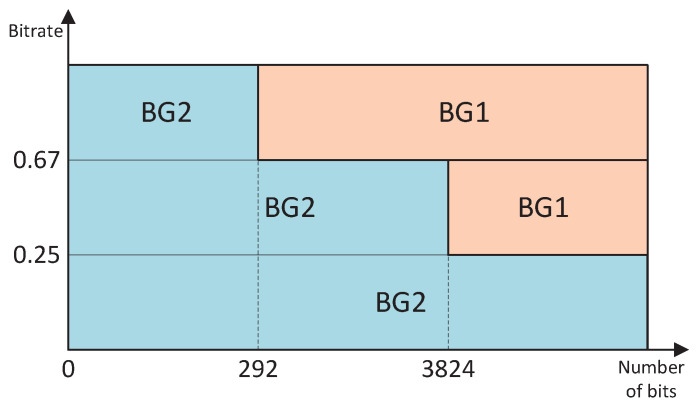
Selection rules for the Base Graph.

Each code block is LDPC-coded to satisfy the checksum equation. The Base Graph (BG) used for LDPC is divided into BG1 and BG2. As shown in Figure 2, when the number of bits to be coded is ≤292 (or the number of bits to be coded is ≤3824 and the code rate is ≤0.67 or ≤0.25), LDPC BG2 is used; for the rest of the cases, it is LDPC BG1. The encoding bits corresponding to the first two columns of the LDPC code base matrix (including BG1 and BG2) are punctured and sent to the rate matching module. After rate matching, bit interleaving is performed to counteract channel burst errors, rearranging the bit sequence to disperse error correlations.

The demodulation and decoding process of LDPC is based on the Belief Propagation (BP) algorithm, which updates the soft information between the Variable Node (VN) and Check Node (CN) through an iterative message passing mechanism, and it then finally converges to the Maximum A Posteriori Probability (MAP) estimate. The log-likelihood ratio (LLR) is typically used as a soft information metric parameter that is adopted by the receiving end, and it is accumulated during retransmission. The larger the absolute value of the LLR, the higher the reliability of the corresponding bit. The QAM demodulation equation for MLC is as follows:

(4) $$\begin{array}{cc} {\mathrm{LLR}}_{k}\left(x,y\right)= & \ln\frac{P(x_{k}=0|y)}{P(x_{k}=1|y)}\\ = & \ln\frac{\sum_{x\in C_{k}^{0}}P\left(y|x\right)}{\sum_{x\in C_{k}^{1}}P\left(y|x\right)}\\ = & \ln\frac{\sum_{x\in C_{k}^{0}}\exp\left(-\frac{{\left|y-x\right|}^{2}}{N_{0}}\right)}{\sum_{x\in C_{k}^{1}}\exp\left(-\frac{{\left|y-x\right|}^{2}}{N_{0}}\right)}. \end{array}$$

where:

- $C_{k}^{0}$ and $C_{k}^{1}$ denote the set of codewords whose *k*th bit is 0 and 1, respectively.
- $N_{0}$ is the noise power spectral density.
- *x* is the QAM symbol sent, *y* is the receiver’s estimate of the complex signal, and ${\left|y-x\right|}^{2}$ denotes the square of the Euclidean distance between *y* and *x*.
- $LLR_{k}$ represents the LLR output corresponding to the kth bit in the QAM symbols.

In the simulation of this scheme, in order to simplify the design, only the scenario where a TB contains only one LDPC code block was analyzed, and the code block segmentation, CB CRC, and scrambling links were omitted. Meanwhile, a direct comparison between the transmitter and the receiver was used instead of CRC checksums for error detection to eliminate unnecessary interference factors. And since the function exp(−x) decreased rapidly with increasing *x*, as shown in Equation (5) (approximating the LLR), the impact on performance was essentially negligible.

(5) $$\begin{array}{cc} {\mathrm{LLR}}_{k}\left(x,y\right) & \approx \ln\frac{\underset{x\in C_{k}^{0}}{\max}\exp\left(-\frac{{\left|y-x\right|}^{2}}{N_{0}}\right)}{\max_{x\in C_{k}^{1}}\exp\left(-\frac{{\left|y-x\right|}^{2}}{N_{0}}\right)}\\ \; & =\frac{1}{N_{0}}\left(\min_{x\in C_{k}^{1}}{\left|y-x\right|}^{2}-\min_{x\in C_{k}^{0}}{\left|y-x\right|}^{2}\right), \end{array}$$

where:

- The approximation is based on the Max-Log approximation, with the maximum operation replacing the summation operation.
- The min operation derives from the monotonicity of the exponential function: $\ln(\max e^{a_{i}})=\max a_{i}$.
- The negative sign reverses the direction of the polar operation: ${\max(-|y-x|}^{2}{)=-\min(|y-x|}^{2})$.

And the other symbol definitions are consistent with the original LLR formula.

The bit stream at the LDPC layer is demodulated at the receiver by first performing a debit interleaving and rate matching operation. The deinterleaving is the inverse of the bit interleaving at the transmitter side. The LDPC decoding uses the Log-MinSum algorithm in the logarithmic domain [31]. The process is as follows.

- VN initialization:
  (6) $$L^{\left(0\right)}\left(q_{ij}\right)=\ln\left(\frac{q_{ij}^{\left(0\right)}\left(0\right)}{q_{ij}^{\left(0\right)}\left(1\right)}\right)=\ln\left(\frac{P_{i}\left(0\right)}{P_{i}\left(1\right)}\right)=L\left(P_{i}\right)\left(={\mathrm{LLR}}_{i}\right).$$
- Iterative decoding is performed:
  (7) $$L^{\left(l\right)}\left(r_{ji}\right)=\alpha \cdot \underset{\mathrm{Sign}\;\mathrm{term}}{\underset{︸}{\prod_{\begin{array}{c} i^{\prime }\in V_{j}\\ i^{\prime }\neq i \end{array}}\mathrm{sign}\left(L^{\left(l-1\right)}\left(q_{i^{\prime }j}\right)\right)}}\cdot \underset{\mathrm{magnitude}\;\mathrm{term}}{\underset{︸}{\min_{\begin{array}{c} i^{\prime }\in V_{j}\\ i^{\prime }\neq i \end{array}}\left|L^{\left(l-1\right)}\left(q_{i^{\prime }j}\right)\right|.}}$$
  Equation (7) is the formula for the CN update, where α is a normalization factor used to improve the accuracy of the Min-Sum algorithm with a typical value of α∈[0.5,1] (around 0.8 is usually considered optimal [32]).
  (8) $$L^{\left(l\right)}\left(q_{ij}\right)=\underset{L(P_{i})\;(\mathrm{initial}\;\mathrm{LLR})}{\underset{︸}{\ln\left(\frac{P_{i}\left(0\right)}{P_{i}\left(1\right)}\right)}}+\underset{\sum L^{\left(l\right)}\left(r_{j^{\prime }i}\right)\;(\mathrm{calibration}\;\mathrm{node}\;\mathrm{message})}{\underset{︸}{\sum_{\begin{array}{c} j^{\prime }\in C_{i}\\ j^{\prime }\neq j \end{array}}\ln\left(\frac{r_{j^{\prime }i}^{\left(l\right)}\left(0\right)}{r_{j^{\prime }i}^{\left(l\right)}\left(1\right)}\right)}}.$$
  Equation (8) is the formula for the VN update.
  (9) $$L^{\left(l\right)}\left(q_{i}\right)=\underset{L(P_{i})(\mathrm{initial}\;\mathrm{LLR})}{\underset{︸}{\ln\left(\frac{P_{i}\left(0\right)}{P_{i}\left(1\right)}\right)}}+\underset{\sum L^{\left(l\right)}\left(r_{j^{\prime }i}\right)(\mathrm{Calibration}\;\mathrm{Node}\;\mathrm{Message})}{\underset{︸}{\sum_{j^{\prime }\in C_{i}}\ln\left(\frac{r_{j^{\prime }i}^{\left(l\right)}\left(0\right)}{r_{j^{\prime }i}^{\left(l\right)}\left(1\right)}\right)}}.$$
  Equation (9) is the formula for a VN a posteriori computation. Equation (10) is the hard judgment rule.
  (10) $${\hat{v}}_{i}=\left\{\begin{array}{cc} 0, & \mathrm{if}\;\underset{\mathrm{posteriori}\;\mathrm{LLR}}{\underset{︸}{L^{\left(l\right)}\left(q_{i}\right)}}>0\\ 1, & \mathrm{else} \end{array}\right..$$
- Iteration termination:
  (11) $$H{\hat{v}}^{⊤}=0.$$

Equation (11) indicates that the LDPC self-check is correct. If Equation (11) holds or reaches the pre-set maximum number of iterations, then the iteration is terminated and the decoded results are output; otherwise, go back to the first step to continue the iteration.

The processed LDPC and BCH layer bit streams are added to the NC layer uncoded bit stream, the three streams are combined for scrambling, and the role of scrambling in a 5G system is mainly to randomize the interference in small intervals and to avoid the possible occurrence of long ‘0’ or long ‘1’ sequences. After scrambling, the data are subjected to MLC constellation mapping.

### 3.2. Bit Rearrangement

The MLCM scheme employs QAM mapping at the transmitter side, which widens the gap between bit channel capacities due to its layered nature. The data are mapped to the constellation points in a certain order during the initial transmission, and they are still mapped in that order during the retransmission. This mapping method results in certain bits being received with significantly higher SNRs than others, depending on their position. After several retransmissions, the difference in the received SNRs between bits at different locations becomes progressively larger. In this case, the uneven SNRs of the received bits at the receiver affects the decoding performance and causes a loss of system gain.

This scheme proposes a bit rearrangement operation at the transmitter during retransmission. The meaning of rearrangement is that the bits in the position of high SNRs at the initial transmission are placed in the position of low SNRs at the time of retransmission. Take the 256QAM shown in Table 1 as an example, the 8 bits of the QAM symbols (a,b,c,d,e,f,g,h) correspond to $i_{0}q_{0}i_{1}q_{1}i_{2}q_{2}i_{3}q_{3}$ at the time of initial transmission, where ‘ab’ bit has the highest SNR, ‘cd’ is the second highest, ‘ef’ is the third highest, and ‘gh’ is the lowest. During retransmission, the 8 bits are reordered. ‘hgfedcba’ corresponds to $i_{0}q_{0}i_{1}q_{1}i_{2}q_{2}i_{3}q_{3}$. At this time, the ‘gh’ bit has the highest SNR, ‘ef’ is next, then ‘cd’ again, and ‘ab’ is the lowest. By the bits rearrangement, the SNR of the bits at each position after retransmission is uniform, and a certain diversity gain can be obtained.

**Figure 3:**
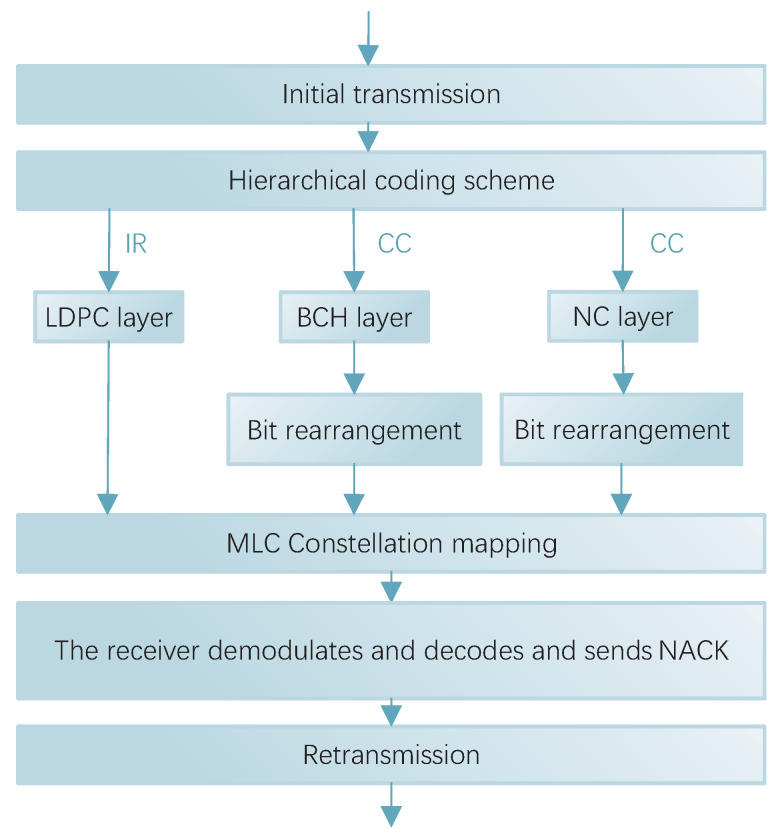
Bit rearrangement schematic.

As shown in Figure 3, since bits rearrangement is the swapping of the corresponding positions in the initial and retransmission, the same length of bits need to be sent for both retransmission and initial transmission. Therefore, it is only useful for the CC method. In this scheme, it was used on the BCH layer and the NC layer.

### 3.3. CC Retransmission Scheme

It can be seen that the key in the CC method lies in the soft information merging mechanism. In the CC method, the transmitter sends a data packet to the receiver. Upon receipt, the receiver first performs a cyclic redundancy check (CRC) on the packet. If correct, an Acknowledgement (ACK) signal is sent to the transmitter. If an error is detected, error correction is first attempted. If unsuccessful, a Negative Acknowledgement (NACK) signal is sent to the transmitter, and the soft information for the packet is cached. The transmitter receives and retransmits the same packet according to the instruction. The receiver receives the new packet and demodulates the information bits to obtain the LLR of the bits. The result is soft merged with the previously cached result. A synthesized signal with a higher SNR is formed, and then decoding is attempted again.

In this approach, to improve reliability by transmitting the same packets with a soft combining mechanism, since each retransmission is a copy of the original transmission, the received Eb/N0 (energy per information bit divided by the noise spectral power density) is increased for each retransmission, improving the probability of correct decoding. The redundant version of the coded bits remains the same for each transmission, as does the puncturing pattern. The receiver uses the current code block and all previous HARQ transmissions to decode the information bits. This process can be repeated until the maximum number of retransmissions or successful decoding is reached. Although the performance gain of the CC method is positively correlated with the number of retransmissions, it is not possible for the system to retransmit endlessly, so the maximum number of retransmissions is usually set to be three to four. When the maximum number of retransmissions is reached, the process is reset and continues to retransmit the same block of code (which is considered a new block).

In BCH coding, the generation of the check digit is realized by generating polynomial division. Equation (12) is the process of calculating the check digit:

(12) $$x^{n-k}b\left(x\right)=a\left(x\right)g\left(x\right)+r\left(x\right),$$

where:

- b(x) is the message code polynomial, where times less than *k*, $x^{n-k}b\left(x\right)$ are the message polynomial b(x) shifted left by n−k bits.
- g(x) is the generating polynomial for the (n,k) BCH code with an n−k number of check symbols.
- a(x) is the quotient polynomial.
- r(x) is the residue polynomial, where the degree of the residue polynomial r(x) is less than n−k.

Equation (13) is the final code word polynomial obtained:

(13) $$c\left(x\right)=x^{n-k}b\left(x\right)+r\left(x\right).$$

This process ensures that c(x) is divisible by g(x), satisfying the algebraic structure property of BCH codes. The method is a cyclic code coding scheme constructed on the basis of finite field algebra theory [33].

As shown in Equation (12), the BCH codes used in the BCH layer are based on the algebraic construction of a finite field, whose parameters such as code length *n*, information bits *k*, and error correction capability *t* are strictly limited to the roots of the generating polynomial g(x). The code rate has to be adjusted by re-selecting g(x), and BCH codes with different code rates cannot share the same algebraic framework. If the code rate is to be changed forcibly, the structure of the system will be too complicated and there will be a loss of error-correction performance. The uncoded bit stream at the NC layer, on the other hand, does not contain redundancy per se and cannot be adapted to the code rate by changing the coding structure. Therefore, both the BCH layer and NC layer use CC retransmission, and it is sufficient to send the same packet for both initial transmission and retransmission.

When the BCH layer and the NC layer perform the CC method, the transmitter sends BCH-encoded or non-encoded message bits in the initial transmission, and the receiver receives them and then checks them for errors. If an error is detected, error correction is attempted. If error correction is not possible, the demodulated LLR is stored in the cache and a NACK is sent back to the transmitter. After receiving the retransmission signal, the transmitter performs a bit-reverse-ordering operation on the bits in order to perform a rearrangement operation. Then, it retransmits them again. The transmitter receives and demodulates the LLR and combines it with the LLR of the initial transmission in the cache end; it then decodes on this basis, and the receiver verifies whether the received data are correct or not. This allows the system to benefit from retransmission and reordering.

### 3.4. IR Retransmission Scheme

The core idea of the IR method is to gradually increase different redundancy check bits during multiple retransmissions. The receiver, then, has enough accumulated redundancy information for more efficient error correction decoding. Through the flexibility of the coding configuration structure, the equivalent code rate of the IR method during retransmission is gradually reduced with increases in the redundant information, thus significantly improving the system throughput under poor channels.

**Figure 4:**
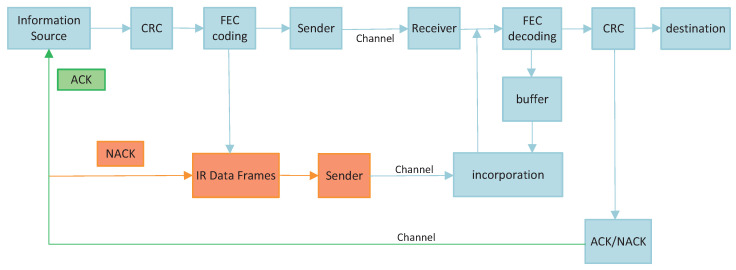
Schematic diagram of the IR method.

The schematic diagram of the IR method is shown in Figure 4. The specific process is that the initial transmission is channel-coded with a code rate of $R_{1}=k/n_{1}$ for the information bits, where *k* is the length of the information bits and $n_{1}$ is the length of the coded block for the initial transmission. If the transmission fails, the LLR is cached and returns a NACK signal to the transmitter. On initial retransmission, the transmitter encodes at the code rate of $R_{2}=k/\left(n_{1}+n_{2}\right)$ according to the preset rules. At this point, the redundancy version changes and only the additional $n_{2}$ parity bits are transmitted. However, the message cannot be recovered by parity bits alone. The receiver needs to decode the newly received redundant bits jointly with the previously cached LLR, and, evidently, $R_{2}<R_{1}$. This time has a lower code rate and thus enhances the error correction capability. This process can be performed iteratively until the decoding is successful or the maximum number of retransmissions is reached.

Since only partial check information is sent during each retransmission, IR requires fewer transmission resources for the same number of retransmissions, thus offering significant advantages in terms of spectrum efficiency. Each retransmission reduces the effective bit rate to assist in decoding information bits, thereby increasing the probability of reliable decoding. Therefore, compared to the CC method, additional coding benefits can be obtained. However, due to the varying redundant bits in each retransmission, the bit length changes after retransmission. Only coding methods with bit rate compatibility can use the IR method. LDPC codes are typical bit rate compatible codes, so the LDPC layer in this scheme adopts the IR method.

An encoding method with rate compatibility must allow different redundancy versions (RVs) to be generated by selecting check bits from the parent code as needed without redesigning, that is, the set of check bits of any subcode must be a subset of the parent code, and the lower code rate version contains all the check bits of the higher code rate version [34]. LDPC codes achieve different code rates by puncturing in the same codeword (base code). The bit positions read from LDPC codes must be integer multiples of the spreading factor *z* to achieve rate matching.

Specifically, the encoding bits corresponding to the first two columns of the LDPC basis matrix are to be punctured, i.e., the first 2z bits are punctured and not transmitted over the air interface, where *z* is the expansion factor, the LDPC code defines eight sets of expansion factors $z=a\times 2^{j}\left(j=0,1,\ldots ,7\right)$, the value of *z* is a positive integer within 2 ≤ *z* ≤ 384, α∈{2,3,5,7,9,11,13,15}, and multiple choices can provide different information block lengths. The punctured bit stream is fed into a circular cache, and the desired length is selected from the starting point of each retransmission. The checksum bits for initial and retransmission can be seamlessly cascaded to form a unified checksum matrix. There is also no need to redesign the decoder, and a unified decoding framework can be used with significantly reduced system complexity.

Take the example of retransmitting once, the first 2z bits of the LDPC code are punctured in the initial transmission, so the *n* bit positions of the initial transmission start after the punctured bits; as such, the initial transmission starts at 2z+1. The retransmission starts at *n* bits after the initial transmission, and the retransmission starts at 2z+1+n. The initial transmission is an (n,k) code, and, after the retransmission, it becomes an (n+n,k) code. The combination of initial and retransmission makes the length *n* of the bit sequence larger, but the message bit length *k* remains the same, thus decreasing the code rate R=k/n. Each retransmission reduces the effective code rate and gives an additional net coding gain due to the reduced code rate. Optimal IR performance is obtained by this retransmission and initial transmission succession.

### 3.5. Demodulation and Decoding

The demodulation and decoding at the receiver is the inverse process of modulation and coding at the transmitter. In this MCLM scheme, the demodulation and decoding at the receiver is carried out in a multi-level series. The layer with the lowest SNR is demodulated and decoded first. Then, the layer with the second lowest SNR is demodulated, etc. In this multi-level decoding architecture, the decoding result of the previous level passes as a priori information to the next level of the demodulator. The result of decoding at each level has an impact on the decoding process at the next level, creating a serial structure. This structure enables the a priori information to be upgraded level by level, gradually improving the accuracy of demodulation and decoding.

In this MLCM-HARQ scheme, it is demodulated to decode the LDPC layer first, then the BCH layer, and finally the NC layer. If each layer is related, then, in the demodulation and decoding process, as long as the receiver checks that there is an error in the data, it sends a feedback signal NACK to the transmitter. The transmitter will start sending the data again, and the scheme needs to set the maximum number of retransmission times *M* to prevent the system from retransmitting indefinitely.

In the case of layer independence, when the code words of the first layer are correctly decoded, they can be placed in the temporary storage space before continuing to decode the next layer. If there is an error in decoding the second layer, the result of the first layer can be directly extracted from the storage space during retransmission. There is no need to repeat the demodulation and decoding steps of the first layer. Layer independence reduces the system workload compared to layer correlation. It can effectively utilize the channel resources, which has a certain improvement on the system performance.

**Figure 5:**
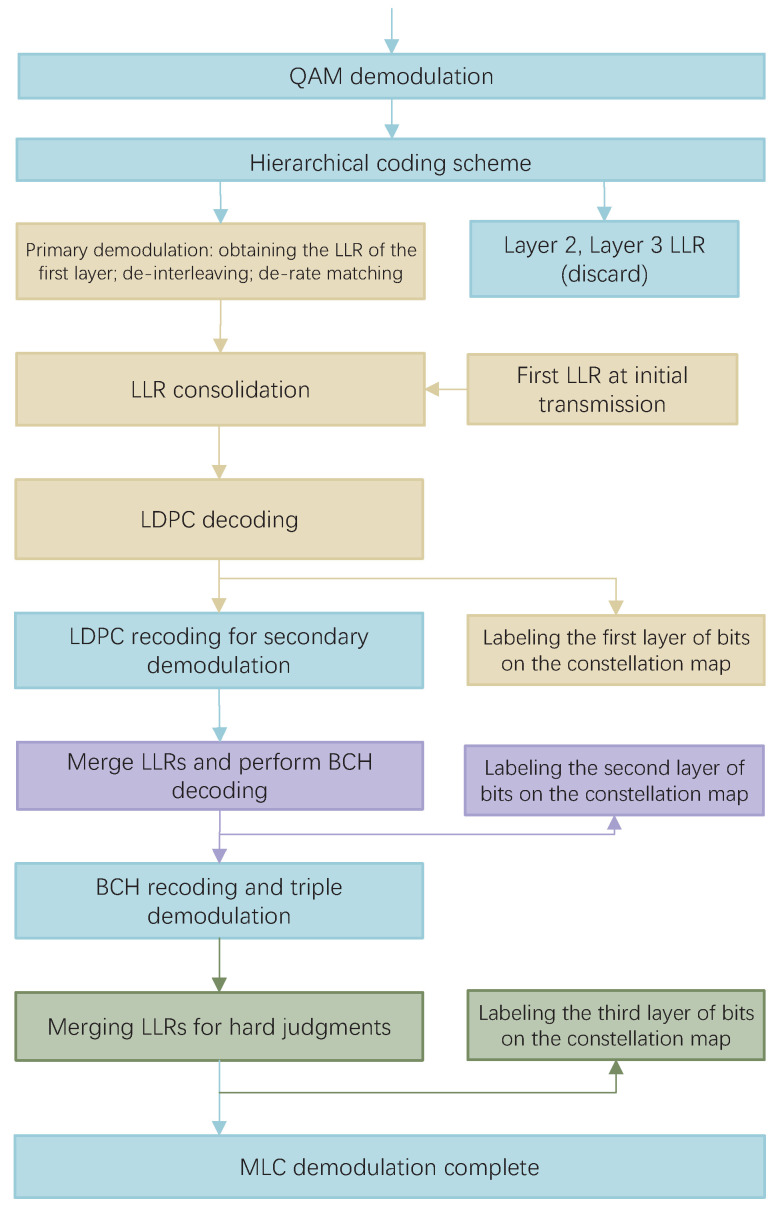
Flowchart of the demodulation and decoding at the receiver of the IR + CC scheme.

The flowchart of the specific steps is shown in Figure 5. The per-layer bitstream aggregate is mapped to the symbol *S*. The receiver receives and performs QAM demodulation on *S* to obtain the LLR of each bit. The LLR of the BCH and NC layers are discarded, and the LLR of the bit streams of the LDPC layer are then calculated first. The result is subjected to a de-bit interleaving and de-rate matching operation. The LDPC layer retransmission is combined with the LLR obtained from the initial transmission, and then LDPC decoding is performed on the combined result. The code word that has been correctly calibrated by the receiver is used as the first layer bit. This code word has a much lower actual code rate and provides some net coding gain. After calibration on the constellation diagram, LDPC encoding is performed again for signal reconstruction. Based on this, a secondary demodulation is performed to merge the initial and retransmitted LLR of the BCH layer, and then BCH decoding is performed. The code word is judged to be correct and, if it is correct, it is used as the second layer bit. On the basis of the first layer, it continues to be calibrated in the constellation diagram. Next, BCH recoding is performed and three times demodulation is performed to obtain the third layer of bits. The third layer is marked in the constellation diagram, and the MLC demodulation is complete.

## 4. Simulation and Analysis

This simulation was performed in MATLAB R2023b. In the simulation, the initial transmission, primary retransmission, and secondary retransmission, with the maximum number of retransmissions set to two, were simulated. BICM was set as the comparison scheme. A visual analysis was then performed of the bit error rate and the block error rate curves of BICM and MLCM.

MLCM adopts a three-tier hierarchical structure comprising LDPC, BCH, and NC layers. Specifically, we demonstrated the performance simulation of MLCM-HARQ using 256QAM404 for a high bit rate (R_total=0.925) and 256QAM440 for a low bit rate (R_total=0.667). In the name, QAM denotes the modulation scheme, 256 represents the modulation order, and the three subsequent digits indicate the bit ratio allocated to the three coding layers: the first digit represents the bits for the LDPC layer, the second digit represents the bits for the BCH layer, and the third digit represents the bits for the NC layer. Specifically, 404 indicates a bit ratio of 4:0:4 for the LDPC layer, the BCH layer, and the unencoded layer, respectively; meanwhile, 440 indicates a bit ratio of 4:4:0 for the LDPC layer, the BCH layer, and the unencoded layer, respectively. Compared to the decoding complexity of LDPC, the decoding complexity of BCH can be neglected in certain specific code lengths and bit rates. Under this MLCM configuration, 50% of the information bits undergo LDPC encoding/decoding, while 50% of the bits are either unencoded or encoded using BCH. Therefore, the encoding/decoding complexity of MLCM is generally reduced to approximately half that of BICM.

To achieve a transmission with an R_total=0.925 code rate, a corresponding simulation parameter list was constructed, as shown in Table 2.

In the 256QAM404 hierarchical structure, in order to achieve the code rate and hierarchical requirements, we first set the total code rate to R_total=0.925, the total input bit length to K_total=8192, and the total output bit length to Ncb_total=8856.

The input bit length and output bit length of BICM were then equal to the total input bit length and total output bit length, that is, K_BICM=K_total=8192 and Ncb_BICM=Ncb_total=8856.

Then, according to the layered structure, Ncb_total was divided into eight parts, and the NC layer and LDPC layer each took four parts. Then, the number of output bits of NC layer was set to Ncb_size1=4428 and the number of output bits of LDPC layer was set to Ncb_size2=4428. Since the NC layer was not coded, the number of input bits of NC layer was set tp K_size1=Ncb_size1=4428. The number of input bits of LDPC layer was set to K_size2=K_total−K_size1=3764 and the LDPC layer code rate was set to R_ldpc=K_size2/Ncb_size2=0.85.

After receiving the feedback message NACK, the transmitter first determines whether the maximum number of retransmissions has been reached. If M = 2 is not reached, the retransmission data are sent to the receiver. If the receiver still fails to feedback the ACK signal when the maximum number of retransmissions is reached at each transmission layer, the currently transmitted data are discarded and the transmitter sends new data.

**Figure 6:**
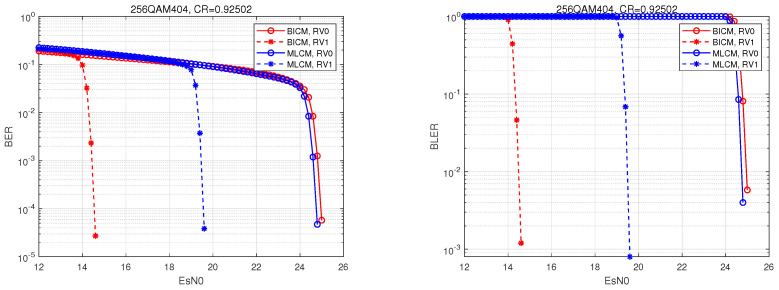
Comparison of the HARQ performance between BICM and MLCM 256QAM404.

The performance curves of the Bit Error Rate (BER) and Block Error Rate (BLER) of the layered scheme 256QAM404 at code rate R=0.925 are shown in Figure 6. In the figure, “MLCM, RV0” represents the performance curve of the initial transmission of MLCM system, and “MLCM, RV1” represents the performance curve of one retransmission of MLCM system. The comparison shows that the primary retransmission brings about 5 dB retransmission gain to the MLCM system, which verifies the practical application effect of the IR + CC retransmission scheme in the MLCM system that was achieved in this paper.

To achieve the transmission of an R_total=0.667 code rate, a corresponding simulation parameter list was constructed, as shown in Table 3.

A combination of the BCH layer and LDPC layer is in the 256QAM440 layered structure. The bit channel SNR of the BCH layer is higher than that of the LDPC layer, so the performance requirements for the coding error correction capability of the BCH layer are not very high. Meanwhile, considering the need to reduce the complexity of the compiled code of the system, BCH codes with t=3 are uniformly used in the BCH layer, specifically the four BCH codes with n=127,255,2047, and 4095. Therefore, the code rate of the BCH layer is fixed, which can be inverted to the code rate of LDPC layer.

The parameters in the single coding structure are set as R_total=0.667, K_total=K_BICM=8194, and Ncb_total=Ncb_BICM=12,282. While, in the hierarchical structure, the Ncb_total is divided into eight parts, the BCH layer and the LDPC layer each take four parts. Then, the number of BCH layer output bits is Ncb_size1=6141 and the number of BCH layer input bits is K_size1=6042. At this time, the total number of coded bits in the BCH coding layer scheme was divided into three groups, and the length of error correction in each group was t=3 bits. The number of LDPC layer output bits was Ncb_size2=6141, the number of LDPC layer input bits was K_size2=2152, and the LDPC layer code rate was R_ldpc=0.35.

**Figure 7:**
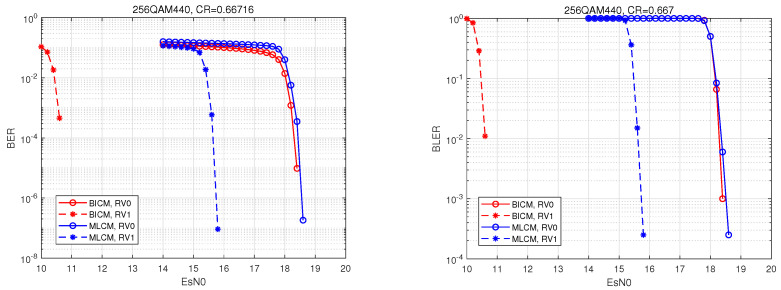
Comparison of the HARQ performance between BICM and MLCM 256QAM440.

At a low bit rate, the corresponding performance curves were recorded, as shown in Figure 7. The comparison showed that a gain of about 3 dB could be obtained for one retransmission at MLCM. This reflects the effectiveness of the IR + CC scheme in this paper in the presence of the BCH coding layer.

As shown in Figure 6 and Figure 7, “BICM, RV0” represents the performance curve of the BICM system for the initial transmission, and “BICM, RV1” represents the performance curve of the BICM system for one retransmission. When observing the curves of BICM during initial transmission, the performance of BICM and MLCM was comparable, and, at that stage, the complexity of MLCM was only half that of BICM. During retransmission, although BICM offered better gain than MLCM, the performance gap between them could be compensated for by increasing the transmit power or by adopting a higher-order modulation, which also provides new insights for our future research. Overall, MLCM can significantly reduce complexity while meeting performance requirements. In terms of balancing complexity and performance, MLCM performs better than BICM, and it is more suitable for applications in complex environments, such as in future 6G technology.

## 5. Conclusions

In this paper, we constructed the MLCM-HARQ retransmission scheme. A theoretical illustration is given in terms of a three-layer structure of MLCM. The three layers of MLCM are coded with the LDPC, BCH, and NC methods, respectively. For the LDPC layer with bit rate compatibility, IR retransmission with additional coding gain is used. For the BCH and NC layers, which are not code-rate compatible, a simple CC retransmission method is used for the fusion of HARQ and MLCM. And the bit rearrangement mechanism is also introduced for the BCH and NC layers so that the SNR of the bits at each position after retransmission is uniformly distributed and a larger diversity gain is obtained.

Taking 256QAM404 and 256QAM440 as examples, we demonstrated the system performance of the IR retransmission at the LDPC layer and the CC retransmission at the BCH and NC layers. It was found that the gain of the LDPC layer in the 256QAM404 layered scenario was 5 dB, and, in the 256QAM440 scenario, the performance could be improved by about 3 dB, which shows that the system performance can be significantly improved at different code rates.

## Figures and Tables

**Table 1:** Schematic diagram of the bit rearrangement scheme.

Transmission Order	64QAM			256QAM			
Bit position	$i_{0}q_{0}$	$i_{1}q_{1}$	$i_{2}q_{2}$	$i_{0}q_{0}$	$i_{1}q_{1}$	$i_{2}q_{2}$	$i_{3}q_{3}$
Initial transmission	ab	cd	ef	ab	cd	ef	gh
Retransmission	fe	dc	ba	hg	fe	dc	ba

**Table 2:** 256QAM404HARQ with R_total=0.925 simulation parameters.

	K_BICM	Ncb_BICM	K_total	Ncb_total	R_total	
BICM	8192	8856	8192	8856	0.925	
	K_size1	Ncb_size1	K_size2	Ncb_size2	R_ldpc	R_total
MLCM	4428	4428	3764	4428	0.85	0.925

**Table 3:** 256QAM440HARQ with CR = 0.667 simulation parameters.

	K_BICM	Ncb_BICM	K_total	Ncb_total	R_total	
BICM	8194	12282	8194	12282	0.667	
	K_size1	Ncb_size1	K_size2	Ncb_size2	R_ldpc	R_total
MLCM	6042	6141	2152	6141	0.35	0.667

## Data Availability

Data are contained within the article.
